# Preparation of zinc hydroxystannate-decorated graphene oxide nanohybrids and their synergistic reinforcement on reducing fire hazards of flexible poly (vinyl chloride)

**DOI:** 10.1186/s11671-016-1403-z

**Published:** 2016-04-12

**Authors:** Tingting Gao, Laicheng Chen, Zhiwei Li, Laigui Yu, Zhishen Wu, Zhijun Zhang

**Affiliations:** National & Local Joint Engineering Research Center for Applied Technology of Hybrid Nanomaterials, Henan University, Kaifeng, 475004 People’s Republic of China; Collaborative Innovation Center of Nano Functional Materials and Applications of Henan Province, Henan University, Kaifeng, 475004 People’s Republic of China; Department of Petrochemical Engineering, Puyang Vocational and Technical College, Huanghe Road, Puyang, 457000 Henan People’s Republic of China

**Keywords:** Zinc hydroxystannate, Graphene oxide, Nanohybrid, Nanoparticle, Flame retardant, Synergistic effect

## Abstract

A novel flame retardant, zinc hydroxystannate-decorated graphene oxide (ZHS/GO) nanohybrid, was successfully prepared and well characterized. Herein, the ZHS nanoparticles could not only enhance the flame retardancy of GO with the synergistic flame-retardant effect of ZHS but also prevent the restack of GO to improve the mechanical properties of poly (vinyl chloride) (PVC) matrix. The structure characterization showed ZHS nanoparticles were bonded onto the surface of GO nanosheets and the ZHS nanoparticles were well distributed on the surface of GO. Subsequently, resulting ZHS/GO was introduced into flexible PVC and fire hazards and mechanical properties of PVC nanocomposites were investigated. Compared to neat PVC, thermogravimetric analysis exhibited that the addition of ZHS/GO into PVC matrix led to an improvement of the charring amount and thermal stability of char residue. Moreover, the incorporation of 5 wt.% ZHS/GO imparted excellent flame retardancy to flexible PVC, as shown by increased limiting oxygen index, reduced peak heat release rate, and total heat release tested by an oxygen index meter and a cone calorimeter, respectively. In addition, the addition of ZHS/GO nanohybrids decreased the smoke products and increased the tensile strength of PVC. Above-excellent flame-retardant properties are generally attributed to the synergistic effect of GO and ZHS, containing good dispersion of ZHS/GO in PVC matrix, the physical barrier of GO, and the catalytic char function of ZHS.

## Background

Recently, graphene (GR) and graphene oxide (GO) are widely used in fabricating advanced composites to provide elevated mechanical performance, excellent electrical properties, etc, due to their outstanding physiochemical properties [1–6]. Among them, many studies also demonstrate that GR and GO are promising flame retardants, which can act as barriers reducing the heat released, insulating against the transfer of combustion gases, and increasing residual char [7–9]. However, for GR and GO, achieving an excellent flame retardancy usually needs a little high loading [10]. Moreover, bare GR and GO possess a propensity to aggregate because of strong van der Waals attractions and π-π attraction between the nanosheets, resulting in decreasing their flame retardancy and deteriorating mechanical properties of polymer matrix. Therefore, the use of GR or GO alone as the flame retardant still remains a challenge.

As is well known, a combination of two or more components can sometimes present a synergistic effect and may impart an excellent flame retardancy to polymers [11]. Particularly, synergistic effects have been extensively found in the family of graphene-based composites in recent years. For instance, the addition of Ce-MnO_2_-GR hybrid sheets can impart excellent flame-retardant properties to an epoxy matrix duo to the synergistic effect [10]. A synergistic effect has also been observed between ZnS and GR in epoxy resin [12]. In addition, the synergistic effects of Ni-Fe-layered double hydroxide/GR hybrids appear in epoxy resin [13]. Surface modification of GR with layered molybdenum disulfide depicts a synergistic reinforcement on reducing fire hazards of epoxy resins [14]. More importantly, the abovementioned modifiers, Ce-MnO_2_, ZnS, Ni-Fe-layered double hydroxide, and MoS_2_, could also inhibit the aggregation of GR and improve the dispersion of GR in polymer matrix, resulting in improved mechanical properties of polymer matrix.

In recent years, zinc hydroxystannate (ZHS) has attracted growing attention owing to significantly improving flame retardancy of poly (vinyl chloride) (PVC). Compared with other inorganic flame retardants, ZHS have outstanding properties, such as low addition, low toxicity, and high efficiency, and will become a useful replacement of the conventional inorganic fillers [15–17]. Unfortunately, ZHS as flame retardant is less competitive in terms of cost and has a poor compatibility like other inorganic fillers in PVC bringing about the deterioration of mechanical properties, which is undesirable for the fabrication of high-performance materials. To overcome this drawback, our previous studies have made efforts to synthesize a composite flame retardant with core-shell structure: nano-ZHS coated by a macromolecule flame retardant, and the nanocomposite flame retardant not only significantly improves flame-retardant and smoke suppression properties of PVC but also does no damage to mechanical properties of PVC [18]. However, this coating technique is relatively complicated. Therefore, a succinct and effective method should be found to improve flame retardancy of ZHS to reduce the usage of ZHS.

Bearing those perspectives in mind, we use ZHS nanoparticles to modify the GO nanosheets to form ZHS/GO nanohybrids utilizing an electrostatic interaction. Herein, ZHS is expected to prevent the aggregation of GO, and GO could also prohibit the aggregation of ZHS, resulting in improving the compatibility between ZHS/GO and PVC matrix. Furthermore, the novel flame retardant, ZHS/GO, was achieved with excellent flame retardancy, using the synergistic effect between ZHS and GO.

## Methods

### Preparation of GO nanosheets

GO nanosheets were prepared from purified natural graphite through the method reported by Hummers and Offeman [19].

### Preparation of ZHS/GO nanohybrids

In a typical procedure, 0.27 g of zinc sulfate heptahydrate and 10.29 mg of as-prepared GO were added to 100 ml of distilled water, followed by sonication for half an hour. Subsequently, 0.28 g of sodium stannate tetrahydrate was dissolved in 20 ml distilled water and added into the above solution, and then the reaction system was maintained at 5 °C for 5 h. Thereafter, 0.27 g of zinc sulfate heptahydrate was added into the above reactant and kept stirring for another 1 h. The final precipitate was collected by filtration and washed several times with distilled water to remove the remaining impurities. In the next step, as-prepared products were dried in an air atmosphere at 80 °C for 12 h. For comparison, bare ZHS nanoparticles were also prepared using the same route without the addition of GO.

### Preparation of ZHS/GO/PVC composite

Preparation of PVC nanocomposite containing 5 wt.% ZHS/GO has similar processing condition with the procedure of DOPO-VTS-ZHS/PVC reported in our literature [20], while DOPO-VTS-ZHS was instead by ZHS/GO in this study. Meantime, for comparison, pristine PVC and PVC nanocomposites with 5 wt.% contents ZHS or GO were also prepared under the same processing conditions.

### Apparatus and experimental method

The morphology and microstructure of as-prepared samples were documented by an X’ Pert Pro MPD X-ray powder diffractometer (XRD), a JEM-2010 transmission electron microscopy (TEM), and an AVATAR360 Fourier transform infrared (FTIR) spectrometer, respectively. Dispersion state of additives in PVC matrix was observed on a scanning electron microscope (SEM) (Nova Nano SEM 450 instrument with an acceleration voltage of 5 kV). Thermogravity analysis (TGA) were conducted on a DSC6200 thermal analyzer at the scanning rate of 10 °C/min. Flame retardancy were carried out by a JF-3 oxygen index meter and a FTT Cone calorimeter, respectively. The tensile strength of the nanocomposites was measured according to the Chinese standard method (GB T1040-92) with a WDW-10D electronic universal testing instrument at the cross head speed of 20 mm/min at 23 °C. Dynamic mechanical analysis (DMA) was performed using a DMA 861e instrument (Metter Toledo Instruments Inc., Swiss Confederation) at a fixed frequency of 10 Hz and a temperature range from 30 to 150 °C at a linear heating rate of 5 °C/min.

## Results and discussion

### Structure characterization of ZHS/GO

**Fig. 1:**
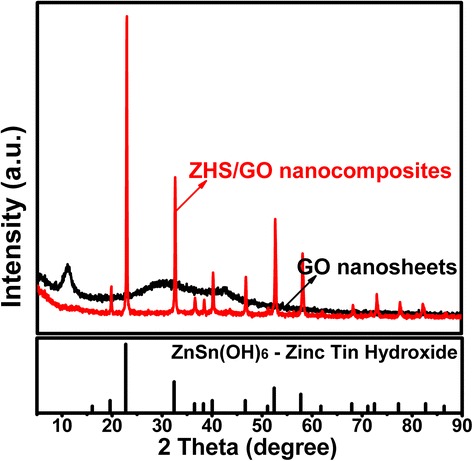
Typical XRD patterns of GO nanosheets and ZHS/GO nanohybrids, showing that the ZHS nanoparticles have become attached on the surface GO nanosheets to form ZHS/GO nanohydrids. The reason is that the crystal growth of ZHS between the interlayers of GO would destroy the regular layer stacking of GO to lead to the disappearance of the diffraction peaks of GO in ZHS/GO nanohybrids

In Fig. 1, the XRD patterns of ZHS/GO exhibit prominent peaks, which can be indexed as the hexagonal structure phase of ZHS. However, no signal for any other phases of GO could be detected in the ZHS/GO nanohybrids. It may be because the regular layer stacking of GO is destroyed due to the crystal growth of ZHS between the interlayers of GO, leading to the disappearance of the diffraction peaks for GO. Therefore, XRD results suggest that the ZHS nanoparticles might have become attached to these GO nanosheets.

**Fig. 2:**
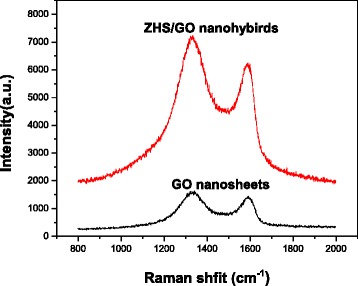
Raman spectra of GO nanosheets and ZHS/GO nanohybrids, which all exhibit two typical peaks of GO at about 1593 cm^−1^ (G band) and 1357 cm^−1^ (D band), resulting in the existence of GO in the ZHS/GO nanohybrids

Because the regular layer stacking of GO is destroyed, there is no any XRD signal of GO in resultant ZHS/GO. Therefore, Raman scattering was used to document the existence of GO. Figure 2 shows Raman spectra of ZHS nanoparticles and ZHS/GO nanocomposites. As shown in Fig. 2, the Raman spectra of both GO and ZHS/GO exhibit two typical peaks of GO at about 1593 cm^−1^ (G band) and 1357 cm^−1^ (D band), which indicates the existence of GO in the ZHS/GO nanocomposites. Combined with the XRD results of ZHS/GO nanocomposites, this further proves that ZHS nanoparticles may be bonded onto the surface of GO.

**Fig. 3:**
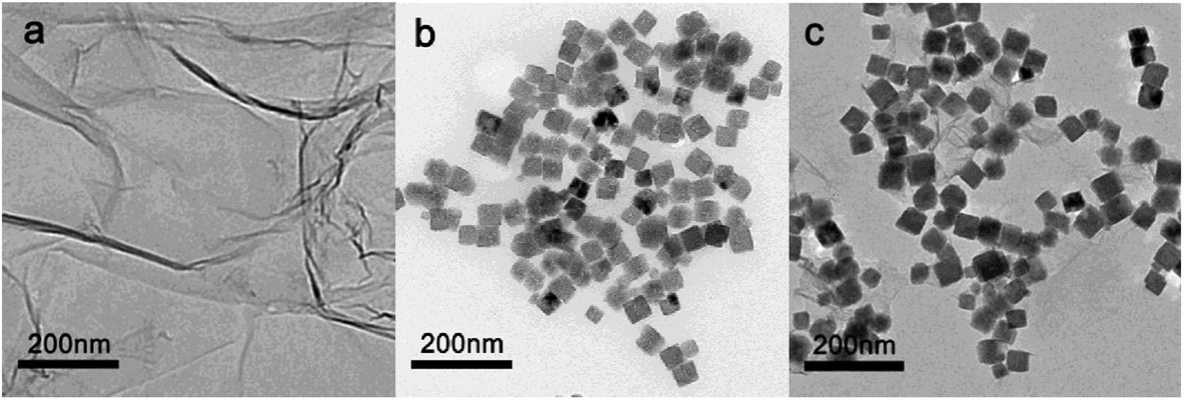
TEM images of **a** GO, **b** ZHS nanoparticles, **c** ZHS/GO nanohybrids. The TEM results show that ZHS nanoparticles successfully bond on the surface of GO. Moreover, the distribution of ZHS nanoparticles on GO nanosheets is uniform and has no obvious aggregation or free nanoparticles are observed

The morphology and microstructure of ZHS/GO nanohybirds are shown in Fig. 3. As can be observed from Fig. 3a, GO nanosheets are highly transparent with folding at the edges, suggesting a very small thickness. Because of their high specific area, GO nanosheets aggregated and formed a stacked graphitic structure when their dispersion solvent was completely evaporated. It is clearly shown in Fig. 3b that the diameter of pristine ZHS nanoparticles is in the range of 40–50 nm. The TEM image of ZHS/GO nanocomposites (Fig. 3c) clearly illustrates that ZHS nanoparticles are distributed on the surface of GO nanosheets. The distribution of ZHS nanoparticles on GO nanosheets is uniform and has no obvious aggregation or free nanoparticles are detected. Furthermore, the TEM picture shows that the diameter of the ZHS nanoparticles is in the range of 50–60 nm, this may be because GO nanosheets facilitate the nucleation of ZHS to lead to a bigger diameter.

**Fig. 4:**
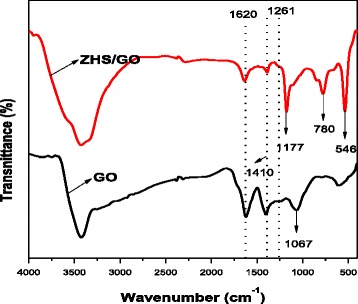
FTIR spectra of GO nanosheets and ZHS/GO nanohybrids, in which the weaker peaks of oxygen-containing functional groups, the new peaks of SnO and ZnO stretching, and the disappeared characteristic peak indicate that ZHS nanoparticles are successfully loaded on the large surface of GO during the electrostatic assembly process thereby affording ZHS/GO nanohybrids

Figure 4 shows FTIR spectra of GO nanosheets and ZHS/GO nanohybrids. As to GO nanosheets, a strong and broad absorption at 3400 cm^−1^ is attributed to O–H stretching vibration. The absorption peak of GO nanosheets at 1620 cm^−1^ due to the C=C stretching vibration, the one at 1410 cm^−1^, is attributed to the tertiary C-OH group, and those at 1261 and 1067 cm^−1^ are assigned to the C–O stretching vibration of –COOH and C–OH groups situated at edges of GO nanosheets [21]. Similarly, after the electrostatic assembly of GO and ZHS, the above stretching of groups situated at GO sheets also appears in ZHS/GO FTIR spectra. However, all the absorption peaks of oxygen-containing functional groups are weaker than those in GO nanosheets. Besides, ZHS/GO shows two peaks at about 780 and 546 cm^−1^, which are assigned to the characteristic stretching vibration of SnO and ZnO stretching, respectively [22]. Moreover, the characteristic peak of C–OH group at 1067 cm^−1^ in ZHS/GO nanohybrids disappears; it is because that the strong absorption intensities of (Sn)O–H at 1177 cm^−1^ covers the C–OH stretching vibration. In conclusion, the weaker peaks of oxygen-containing functional groups, the new peaks of SnO and ZnO stretching, and the disappeared characteristic peak indicate that ZHS nanoparticles are successfully loaded on the large surface of GO during the electrostatic assembly process thereby affording ZHS/GO nanohybrids.

**Fig. 5:**
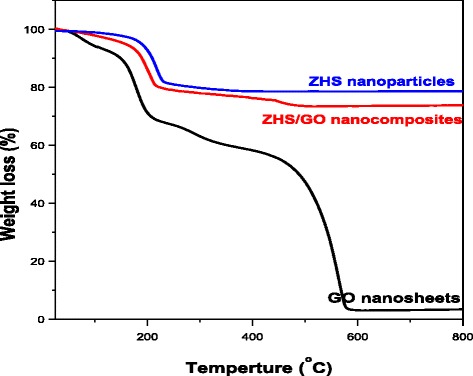
TGA curves of GO nanosheets, ZHS nanoparticles, and ZHS/GO nanohybrids under air condition with raising rate of temperature of 10 °C/min. The mass loss of ZHS/GO is less than that of GO nanosheets and more than that of ZHS nanoparticles, respectively, which further indicates ZHS nanoparticles decorated on the surface of GO nanosheets

TGA curves of GO nanosheets, ZHS nanoparticles, and ZHS/GO nanohybrids are shown in Fig. 5. For ZHS, the mass loss is the lowest, 21 %, which is mainly due to the dehydroxylation of ZnSn(OH)_6_ generating ZnSnO_3_. For GO, in agreement with previous reports in the literature, the main mass loss (32 %) takes place around the 100–300 °C range, corresponding to the decomposition of labile oxygen functional groups present in the material [23]. There is also a mass loss (6 %) below 100 °C, which can be attributed to the removal of adsorbed water and a slower, steady mass loss (56 %) over the whole temperature range between 400 and 600 °C, which can be assigned to the removal of more stable oxygen functionalities. As for ZHS/GO nanohybrids, after ZHS nanoparticles are bonded onto GO, the total weight loss is 22 %, between ZHS and GO, which should be closely related to the dehydroxylation of ZHS and oxidation of GO. It may be the tin compounds can react with the GO in higher temperature making the GO to become stable. All indicated that ZHS nanoparticles are bonded onto the surface of GO.

**Fig. 6:**
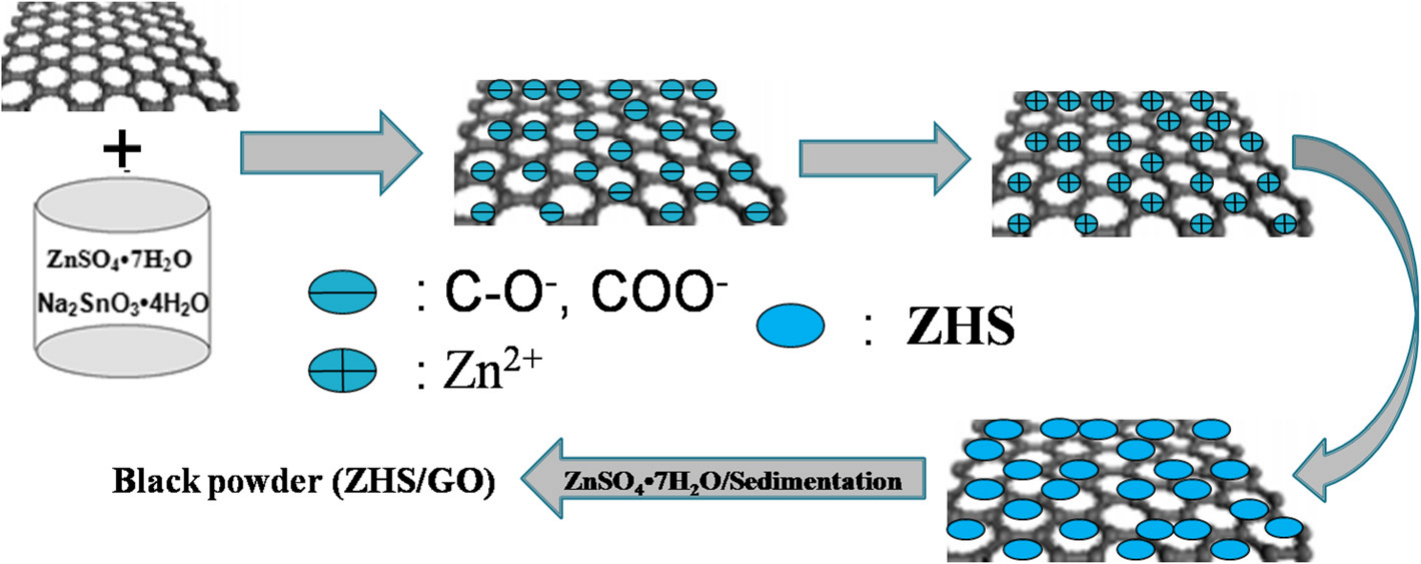
The possible formation process of ZHS/GO nanocomposites. Zn^2+^ cations fist are captured by GO through the electrostatic interactions and then react with SnO_3_^2−^ to form ZHS nanoparticles on the surface of GO

The possible formation mechanism of ZHS/GO is proposed according to the above TEM, XRD, FTIR, and TGA analyses. Figure 6 schematically illustrates the formation mode of ZHS/GO nanohybrids. It is well known that there are considerable oxygen-containing functional groups on the surface of GO nanosheets. At the same time, the GO nanosheets in the pH range of 4 to 11.5 can lead to deprotonation of the oxygen-containing acidic groups, which establishes a negative surface charge density [24]. The large number of the negative charge which contains carboxylate (COO−) anion and phenolate anion (–C–O–) [25] etc. on the surface of GO nanosheets could capture and bond with Zn^2+^ in zinc sulfate solution through electrostatic attraction. Subsequently, the sodium stannate solution was added, the growth of zinc hydroxystannate by way of chemical reaction between Zn^2+^ cation and SnO_3_^2−^ anion. Finally, ZHS nanocrystals further assembled and became ZHS/GO nanostructures driven by the minimization of surface energy.

### Morphology of PVC and its nanocomposites

**Fig. 7:**
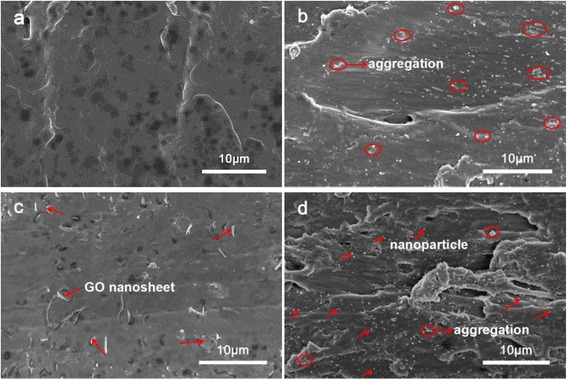
SEM photographs of the fractured sections of **a** PVC, **b** ZHS/PVC, **c** GO/PVC, and **d** ZHS/GO/PVC, respectively, revealing the better dispersion of ZHS/GO in the PVC matrix than ZHS or GO alone in the PVC matrix

SEM is a widely used technique to evaluate the dispersion level of fillers in a polymeric matrix. Figure 7 shows the images of the fractured surfaces for pure PVC and its nanocomposites. The fracture roughness of the polymer materials may demonstrate the dispersion state and interfacial interaction to some degree [26]. For pure PVC, it presents a uniform surface morphology revealing a rather smooth surface (Fig. 7a). Meanwhile, the GO nanosheets agglomerated obviously in the PVC matrix because of the re-stack of them (Fig. 7c). When ZHS nanoparticles are incorporated into PVC, the dispersion of ZHS seems to be uniform; however, some aggregates are still observed (Fig. 7b). Compared to pristine ZHS particles, the dispersion of ZHS/GO nanohybrids were improved (Fig. 7d). Herein, the fracture surfaces of ZHS/GO/PVC are much rougher than those of ZHS/PVC and GO/PVC, indicating the better dispersion and stronger interfacial interaction of ZHS/GO/PVC. The well-dispersed nanoparticles are generally due to the oxygen-containing groups acting as anchoring sites for ZHS on the surface of GO. The results demonstrate better dispersion of ZHS/GO in the PVC matrix than ZHS or GO alone in the PVC matrix, and the dispersion of the nanofillers is crucial for the performance of the nanocomposites.

### Thermal degradation of PVC and its nanocomposites

**Fig. 8:**
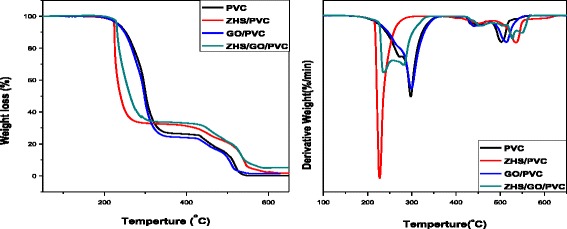
TGA and DTG curves of pure PVC, ZHS/PVC, GO/PVC, and ZHS/GO/PVC under air condition with raising rate of temperature of 10 °C/min. ZHS/GO/PVC nanocomposite presents a little degradation behavior compared to the neat PVC, which is attributed to the combination of the cross-linking effect of ZHS nanoparticles and good mass barrier effects of GO. The temperature at the maximum rate of weight loss at the second stage of decomposition (T_d,max_2) of ZHS/PVC, GO/PVC, and ZHS/GO/PVC were obviously higher than that of PVC. These results revealed that the stability of the residue was enhanced

Figure 8 shows the TGA curves of PVC and corresponding nanocomposites containing 5 wt.% each flame retardant. And some important data are summarized in Table 1. As shown in Fig. 8a, the thermal decomposition of flexible PVC composites is simply divided into two stages. The first stage in the temperature range of 150–380 °C is attributed to the dehydrochlorination of the polymer chain and plasticizer degradation. And the second stage beyond 430 °C mainly contains cyclization of conjugated polyene sequences to form aromatic compounds and oxidation of the unstable char. However, compared with neat PVC, the temperature at the maximum rate of weight loss at the first stage of decomposition (T_d,max_1) of ZHS/PVC nanocomposite was obviously lower than that of PVC, while on the contrary, the T_d,max_1 of GO/PVC was slightly higher than that of PVC. That is because ZHS can catalyze HCl released from PVC and promote the early crosslinking to the PVC compound, while the GO has not the catalytic action. However, for GO/PVC, the physical barrier effect of GO can inhibit the mass and heat loss of polymer; therefore, the T_d,max_1 of GO/PVC was slightly increased. For the ZHS/GO/PVC nanocomposite, it presents a little degradation behavior compared to the neat PVC. These results attributed to the combination of the cross-linking effect of ZHS nanoparticles and good mass barrier effects of GO. The temperature at the maximum rate of weight loss at the second stage of decomposition (T_d,max_2) of ZHS/PVC, GO/PVC, and ZHS/GO/PVC was obviously higher than that of PVC. These results revealed that the stability of the residue was enhanced. To the aspect of the formation of the char, the residues at 650 °C of ZHS/GO/PVC is significantly increased, owning to a catalyze carbonization effect of ZHS and the physical barrier function of GO. Furthermore, from DTG profiles (Fig. 8b), the maximum mass loss rate of ZHS/GO/PVC decreases significantly, suggesting that ZHS/GO nanocomposites function as a barrier to inhibit the mass loss during the thermal degradation process.

**Table 1:** Summary of the parameters of thermal and mechanical properties

Sample	T_d, max_1 (°C)	T_d, max_2 (°C)	Char yield at 650 °C	LOI (%)	Tensile strength (MPa)
PVC	297.2	501.4	0.05	24.5 ± 0.1	11.9 ± 0.4
ZHS/PVC	227.2	536.2	2.0	27.2 ± 0.1	13.4 ± 0.2
GO/PVC	299.3	513.1	1.47	25.9 ± 0.1	13.2 ± 0.2
ZHS/GO/PVC	236.8	527.6	5.1	28.5 ± 0.1	14.3 ± 0.3

*T*_*d, max*_ *1* the peak value of the first-derivative profiles derived from TGA curves, *T*_*d, max*_ *2* the peak value of the second-derivative profiles derived from TGA curves

### Fire hazard evaluated by LOI and cone calorimetry

**Fig. 9:**
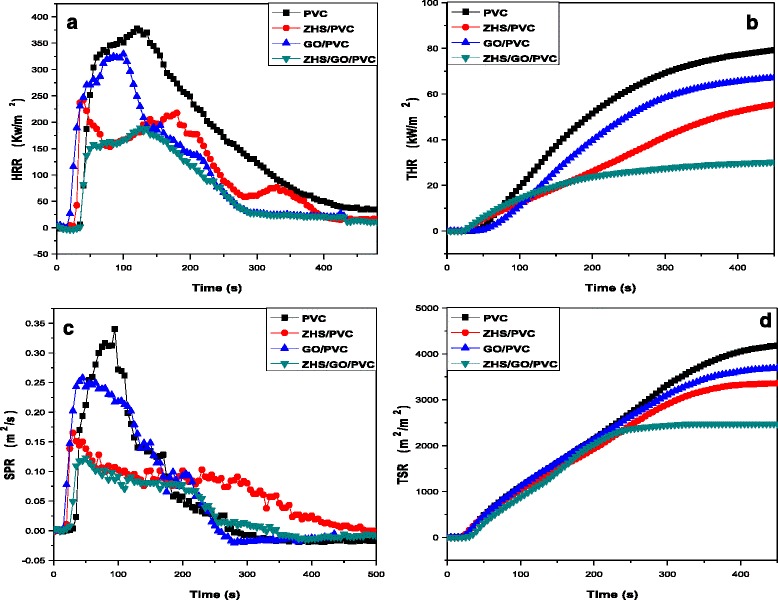
HRR (**a**), THR (**b**), SPR (**c**), and TSR (**d**) versus time curves of PVC and its nanocomposites obtained from cone calorimetry test with a heat flux of 35 kW/m^2^, in which ZHS/GO nanohybrids show the lowest pHRR, THR, SPR, and TSR compared to other nanocomposites, indicating ZHS/GO nanohybrids possess the best flame retardancy to PVC

The LOI values of all nanocomposites are presented in Table 1. The LOI value increases drastically from 24.5 to 27.2 % when the ZHS was added into PVC composites. The LOI value of GO/PVC nanocomposite exhibits an insignificant increase. However, this value is still higher than the neat PVC system, which increased 24.5 to 25.9 %. In contrast, the LOI value of ZHS/GO/PVC nanocomposites improves significantly, namely, from 24.5 to 28.5 % when ZHS/GO was added. The results indicate that ZHS/GO imparts a good flame retardancy on PVC at low filler loadings which are probably due to the shielding effect of GO and the catalyzing charring effect of ZHS (Fig. 9 and Table 2).

**Table 2:** Cone date of PVC, ZHS/PVC, GO/PVC, and ZHS/GO/PVC

sample	pHRR (kW/m^2^)	THR (MJ/m^2^)	TSR (m^2^/m^2^)	AMLR (g/s)
PVC	377.1	83.12	4282	0.36
ZHS/PVC	242.5	59.35	3383	0.20
GO/PVC	327.1	67.66	3737	0.30
ZHS/GO/PVC	185.5	33.45	2467	0.15

*pHRR* the peak heat release rate, *THR* total heat release, *TSR* total smoke release, *AMLR* average mass loss rate

**Fig. 10:**
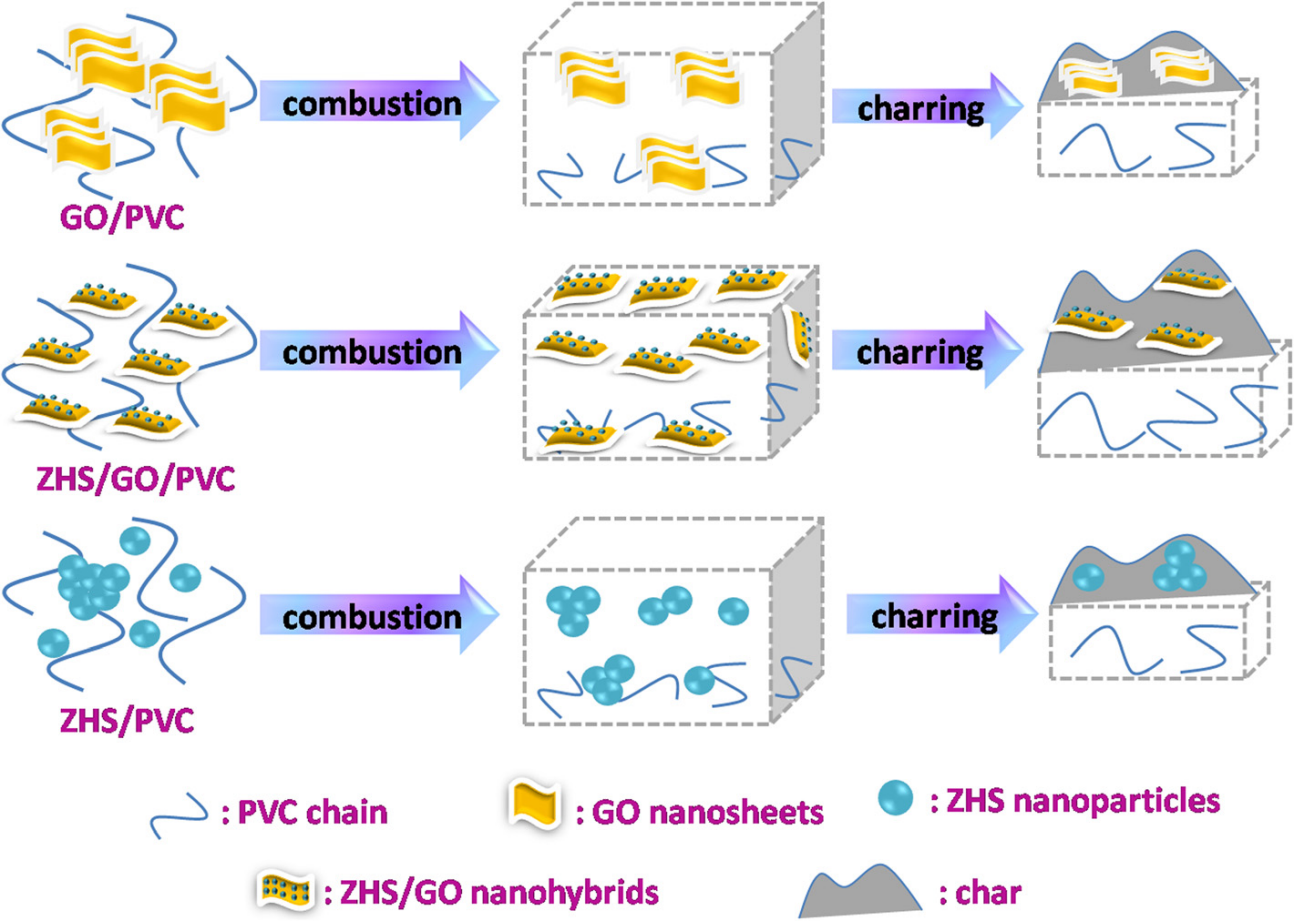
Schematic representation of the mechanism for the improved fire-resistant properties of the ZHS/GO/PVC nanocomposite, showing that the synergistic effect between ZHS and GO can better improve the flame-retardant performance of PVC

The flammability of polymer materials is commonly characterized by cone calorimeter, which is very effective to evaluate their flame-retardant properties under real-world fire conditions. Heat release rate (pHRR), total heat release (THR), smoke production rate (SPR), and total smoke release (TSR) curves for PVC and its composites are shown in Fig. 9, and some important parameters are listed in Table 2. As shown in Fig. 9a, incorporating 5 wt.% ZHS or GO into PVC generates pHRR decrease to 242.5 and 327.1 kW/m^2^, corresponding to a 35.7 and 13.2 % reduction compared to pure PVC, respectively. The pHRR and THR for ZHS/GO/PVC show a further decrease compared to the GO/PVC or ZHS/PVC, which are just 185.5 and 33.45 kW/m^2^ corresponding to a 50 and 59.7 % reduction to pure PVC, respectively. In addition, the samples having ZHS/GO nanohybrids show the lowest SPR, TSR, and AMLR compared to other nanocomposites, which conforms well to the TGA, pHRR, and THR results. The superior flame retardancy of ZHS/GO/PVC mainly includes three reasons: the barrier effect of GO, which retards the permeation of heat and the escape of volatile degradation products; the catalytic carbonization effect of ZHS to form more and better chars, which decrease the flammability of PVC; and the ZHS-contained Zn element should play an important role to eliminate volatile organic compounds and toxic gases during combustion reactions [27]. The proposed mechanism for the improved fire-resistant properties of the ZHS/GO/PVC nanocomposite is illustrated in Fig. 10. During the combustion process, the ZHS nanoparticles loaded on GO prevent the GO from re-stacking and result in the good dispersion. Meanwhile, GO can function as a physical barrier to absorb degradation products and extend contact time with ZHS catalyst. Degradation products continually collect on the GO, which serves as a template for the formation of char. Furthermore, the degradation products are catalytically converted into char by the combination of the physical barrier effect of GO and the catalytic effect of ZHS.

### The mechanical properties of PVC and its nanocomposites

**Fig. 11:**
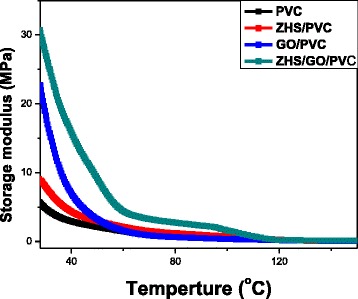
Storage modulus curves of PVC and its nanocomposites as a function of temperature, showing ZHS/GO/PVC nanocomposite has the high-storage modulus, which is attributed to the synergistic reinforcement effect of ZHS/GO nanohybrids in polymer matrix

The values of tensile strength of PVC and its nanocomposites with different additives are presented in Table 1. Pure PVC shows a high tensile strength of 12.9 MPa. When ZHS and GO are added, the tensile strength at break of composite is increased to 13.4 and 13.2 MPa, respectively. In addition, the tensile strength at break of ZHS/GO/PVC nanocomposite increases from 12.9 to 14.3 MPa. The increase of the tensile strength could be interpreted on the basis of following factors: the strong interfacial adhesion and good dispersion of ZHS/GO in the PVC, resulting in effective load transfer from the polymer to GO [28]; the enhancement of crosslink density is due to the hydroxyl on the surface of nanoparticles [29]; and the viscous energy absorption and crack deflection [30]. Especially, ZHS nanoparticles could prevent the restack of GO and GO could also ensure ZHS nanoparticles remain well dispersed in PVC, thereby enhancing the tensile strength. In addition, the ternary structure could absorb much more energy than the binary nanocomposites [31, 32], namely, the effective synergistic strengthening in ZHS/GO/PVC achieves the high mechanical properties, superior to ZHS/PVC and GO/PVC nanocomposites (Fig. 11).

Dynamic mechanical analysis (DMA) test was performed to determine the reinforcement of ZHS/GO on the dynamic mechanical properties of the PVC nanocomposites. Figure 11 shows the storage modulus of neat PVC and its nanocomposites. It can be seen that the storage modulus of either ZHS/PVC or GO/PVC is increased compared to that of neat PVC within the entire temperature range, which is attributed to the reinforcement effect of additives. Similar with the tensile strength, the storage modulus of ZHS/GO/PVC nanocomposite further increased compared to ZHS/PVC and GO/PVC, which is also attributed to the synergistic reinforcement effect of ZHS/GO nanohybrids in polymer matrix.

## Conclusions

ZHS/GO nanocomposites were successfully synthesized. The morphological characterization showed that the synthetic ZHS/GO nanocomposites exhibited some independent and separate ZHS nanoparticles are well distributed on the surface of GO. TGA revealed that the ZHS/GO nanocomposites could enhance the residue yield compared with pure GO. The incorporation of 5 wt.% ZHS/GO nanocomposites into PVC led to the improvement of T_d,max_2, char residue, DTG peak value, LOI, and mechanical properties compared to those of pure PVC. Furthermore, the pHRR and THR values for ZHS/GO/PVC were significantly reduced by 50 and 59.7 %, respectively, compared to those of pure PVC. Moreover, the amount of organic volatiles released during the combustion of PVC was significantly reduced after incorporating ZHS/GO. The improved flame retardancy was obtained through the synergistic effect between ZHS nanoparticles and GO nanosheets, resulting from the physical barrier effect acted by GO in combination with the effect of ZHS.
